# Adherence to *Legionella* control regulations and guidelines in Norwegian nursing homes: a cross-sectional survey

**DOI:** 10.1186/s12889-024-18993-x

**Published:** 2024-06-04

**Authors:** Anders Bekkelund, Line Ødegård Angeloff, Ettore Amato, Susanne Hyllestad

**Affiliations:** Department of Zoonotic, Food- and Waterborne Infections, Norwegian Institute of Public Health, Oslo, P.O. box 222, Norway

**Keywords:** Legionella, Risk assessment, Nursing homes, Water systems, Preventive measures

## Abstract

**Background:**

Infection by *Legionella* bacteria is a risk to elderly individuals in health care facilities and should be managed by preventing bacterial proliferation in internal water systems. Norwegian legislation calls for a mandatory *Legionella*-specific risk assessment with the subsequent introduction of an adapted water management programme. The present study investigates adherence to legislation and guidelines on *Legionella* control and prevention in Norwegian nursing homes.

**Methods:**

A cross-sectional survey was distributed to Norwegian municipalities to investigate the status of *Legionella* specific risk assessments of internal water distribution systems and the introduction of water management programmes in nursing homes.

**Results:**

A total of 55.1% (*n* = 228) of the participating nursing homes had performed *Legionella*-specific risk assessments, of which 55.3% (*n* = 126) stated that they had updated the risk assessment within the last year. 96.5% introduced a water management programme following a risk assessment, whereas 59.6% of the ones without a risk assessment did the same. Nursing homes with risk assessments were more likely to monitor *Legionella* levels than those without (61.2% vs 38.8%), to remove dead legs (44.7% vs 16.5%), and to select biocidal preventive treatment over hot water flushing (35.5% vs 4.6%).

**Conclusions:**

This study presents novel insight into *Legionella* control in Norway, suggesting that adherence to mandatory risk assessment in nursing homes is moderate-low. Once performed, the risk assessment seems to be advantageous as an introduction to future *Legionella* prevention in terms of the scope and contents of the water management programme.

**Supplementary Information:**

The online version contains supplementary material available at 10.1186/s12889-024-18993-x.

## Background

The genus *Legionella* includes Gram-negative microorganisms naturally occurring in soil and water bodies. If introduced to buildings through the water supply, it may proliferate in favourable conditions, e.g., water temperatures between 20 °C and 50 °C, stagnant water, and the presence of biofilm [1]. *Legionella* infection is usually acquired by inhaling water aerosols from showers and similar devices, and may cause legionellosis, either as Legionnaires’ disease, a potentially fatal form of pneumonia, or Pontiac fever, a flu-like illness. Aspiration of water is also reported as a major cause of nosocomial legionellosis [2]. Legionellosis is the most frequent cause of waterborne outbreaks worldwide in high income countries [3], with *L. pneumophila* serogroup 1 most commonly associated with infection [4, 5]. Contamination of cooling towers among others have often been reported as the cause of *Legionella* outbreaks [6]. Growth of *Legionella* in water systems are associated with biofilm, thus, preventing biofilm formation is a key strategy to reduce the risk of *Legionella* contamination in domestic water systems [7].

The control and prevention of *Legionella* have been developing since the association between Legionnaire’s disease and water systems was first described in the early 1980s [8]. Several guidelines for the prevention and control of *Legionella* exist [9–11]. Preventive strategies include avoiding water temperatures favourable for *Legionella* growth, reducing water stagnation by regular flushing, and removing pipes without water flow, so-called dead legs, supplemented with monitoring of *Legionella* levels where appropriate [12]. When appropriate, on-site biocidal water treatment, such as copper-silver ionization or chlorine dioxide, is also widely used as a continuous water treatment [13] and has been shown to reduce *Legionella* levels [14, 15]. Several reports highlight the importance of risk-based monitoring and management of *Legionella* [16]. In one review, the authors point out that all preventive methods must be accompanied by a water management programme, as disinfection alone might not be sufficient to eliminate the bacteria once a system is contaminated [17]. This should also include measures beyond monitoring bacterial levels, as the infectious dose is uncertain [18].

Confirmed cases of Legionnaire’s disease in Norway have been registered in the National Surveillance System for Notifiable Diseases since 1977. Few annual cases were registered prior to the 1990s, when an increase occurred, yielding a range of 25–70 cases per year nationwide in the last 30 years. More than half of the cases are normally reported to have been infected abroad [19, 20]. An evaluation of legionellosis surveillance in Norway found that it detects incidence changes for Legionnaire’s disease over time, by place and person, but likely does not detect every diagnosed case due to lack of standardised procedures for *Legionella* testing in hospitals [21].

Following three major outbreaks of Legionnaire’s disease in Norway in 2001, 2005, and 2008, associated with cooling towers and an air scrubber [22, 23], provisions on *Legionella* prevention were included in Norwegian legislation, imposing on all owners of technical installations with the potential to spread *Legionella* to perform a risk assessment and introduce preventive measures [24]. Details are elaborated in national guidelines on the control and prevention of *Legionella*, identifying critical control points for risk assessments, and how to design a subsequent prevention and control programme. According to the guideline [25], a comprehensive risk assessment should encompass activities such as i) a mapping of the entire system, specifically identifying areas with risk of *Legionella* proliferation and spread, e.g., stagnant water, favourable water temperatures, and aerosolization, ii) temperature measurements, iii) *Legionella* monitoring, iv) assessment of existing preventive routines, and v) suggestions for how to proceed with a *Legionella* management programme.

In addition, the general principles of risk-based approach of water safety planning may be considered [26]. Healthcare facilities are of special concern due to the complexity of the water systems and the susceptibility of users to infection [25]. Monitoring of *Legionella* in priority premises was also recently introduced in the revised EU Directive on drinking water [27].

Norwegian nursing homes are part of the general health care system, generally organised and funded at the municipal level, with options for public–private partnership. Municipal health care, including nursing homes, shall provide equal and adequate health care to all inhabitants [28, 29].

Research on *Legionella* in Norway has primarily focused on the identification of sources of outbreaks [9–11] and the detection or characterization of *Legionella* [30, 31]. There is limited research on compliance with guidelines, except for studies from Italy [32–34] and the USA [35]. To the best of our knowledge, the scope of *Legionella* control in Norwegian nursing homes has not been previously studied. To address this knowledge gap on adherence to Norwegian regulations and guidelines on *Legionella* prevention in nursing homes, we carried out a national survey to gain more insight into routines and practice. The aim was to establish an understanding of the extent of *Legionella* prevention and subsequently consider any need for clarification of the guidelines.

## Methods

### Study and survey design

We conducted a cross-sectional survey among municipal nursing homes in Norway. A questionnaire designed to collect data specifically aimed at adherence to provisions and the contents of preventive measures against *Legionella* was distributed by e-mail to all 356 municipalities in Norway in 2020 along with a request to forward it to all nursing homes within their municipality. The rationale for not contacting the nursing homes directly was that contact information is not available for each nursing home. As nursing homes are operated by the municipalities, this was hence the obvious contact gateway. The questionnaire consisted of 57 questions divided into three sections: i) information about *Legionella* risk assessment, ii) details about the water management program, and iii) information about *Legionella* monitoring. After assessing the answers, we included seven questions that were suitable for analysis in the present study.

At the time of the survey, we could potentially reach a total of 866 municipal nursing homes [36]. As a nursing home may comprise several physical locations, we asked the respondent to provide one reply for each location. Duplicate or blank answers, answers that covered several nursing homes, or where it was unclear if more than one location was covered, were excluded from the dataset. Data were analysed with Microsoft Excel. Chi-squared test was used to validate differences between respondents who did or did not perform a risk assessment. We considered a *p*-value of < 0.05 as statistically significant.

## Results

### *Legionella*-specific risk assessments

A total of 449 replies were received from Norwegian nursing homes. Removal of duplicates yielded 414 replies representing one location each, a response rate of 47.8%. The responses covered all 11 Norwegian counties and 247 of 356 municipalities (69.4%), with response rates for nursing homes spanning between 41.6% in the northernmost region and 65.9% in the south.

A total of 55.1% (*n*=228) of the responding nursing homes stated that they had performed a *Legionella*-specific risk assessment of which 55.3% (*n*=126) had updated it within the year preceding the survey, and 18.6% (*n*=77) did not know whether a risk assessment had been performed (Table 1). The nursing homes with a water management programme were asked if they monitor *Legionella* levels, and 61.2% confirmed that they do.

**Table 1:** Summary of the main results for Norwegian nursing homes included in this study

	**n**	**%**
**Participating nursing homes (** *n***=866)**	414	47.8
**Performed a ** ***Legionella*-specific risk assessment (** ***n*=414)**		
Yes	228	55.1
No	109	26.3
Unknown	77	18.6
**Updated risk assessment within the last year (** ***n*=228)**		
Yes	126	55.3
No	81	35.5
Unknown	21	9.2
**Introduced** ***Legionella*** **prevention (** ***n*=414)**		
Yes	330	79.7
No	43	10.4
Unknown	41	9.9
***Legionella*** **monitoring (** ***n*=330)**		
Yes	202	61.2
No	128	38.8

### *Legionella* management programme

330 (79.7%) of the participating nursing homes had introduced a *Legionella* management programme, while 43 (10.4%) facilities had not, and 41 (9.9%) did not know whether a management programme was introduced (Table 1). A total of 96.5% (*n* = 220) of nursing homes with a risk assessment have introduced a *Legionella* prevention programme. This significantly decreases to 59.6% (*n* = 65) for those without a risk assessment (Table 2). A similar significant coherence is evident for other investigated parameters when analysed for whether a risk assessment has been performed or not, such as removal of dead legs (44.7% vs 16.5%) and chemical treatment as the primary disinfection method (35.5% vs 4.6%). No significant difference was observed for hot water flushing as the primary disinfection method (25.9% vs 22.9%).

**Table 2:** Summary of investigated parameters based on whether Norwegian nursing homes have performed a *Legionella* risk assessment

	Performed a risk assessment (*n*=228)		Did not perform risk assessment (*n*=109)		
	n	%	n	%	*p*-value*
**Introduced a Legionella management programme**	220	96.5	65	59.6	<.001
**Monitoring of Legionella levels**	154	67.5	26	23.9	<.001
**Removal of dead legs**	102	44.7	18	16.5	<.001
**Hot water flushing as primary prevention**	59	25.9	25	22.9	0.559
**Continuous chemical treatment as primary prevention**	81	35.5	5	4.6	<.001

*The *p*-value calculated using Chi-squared test with significance level <0.05

All nursing homes performing a *Legionella* risk assessment (*n* = 228) were surveyed with additional questions on what critical control points were included in the risk assessment. Most commonly reported were temperature measurements (*n* = 196; 86.0%) and identification of areas at risk for *Legionella* proliferation (*n* = 185; 81.1%). A total of 49.6% (*n* = 113) reported having assessed aerosol spread potential, and 49.1% (*n* = 112) had contingency plans in case of an outbreak. A total of 54.8% (*n* = 125) monitored *Legionella* levels as part of the risk assessment, while 32.0% (*n* = 73) performed total plate count.

## Discussion

In this study, we investigated adherence to *Legionella* regulations and guidelines in Norwegian nursing homes. To our knowledge, this is the first study addressing this topic in Norway. The responses cover all parts of the country, making the outcome of the study relevant in a national context.

### Adherence to regulations and guidelines

The legally required *Legionella*-specific risk assessment being performed by just over half of responding Norwegian nursing homes is moderate-low, considering that the occupants are at high risk for contracting severe legionellosis. Keeping the risk assessment up to date is emphasized in the Norwegian guidelines, with a recommendation for at least annual updates, or in the event of changes relevant to *Legionella* risk. Only 55% of the nursing homes stated that they had done an update within the preceding year.

Shortage of trained health care professionals is an issue in Norway [37], especially in rural areas, and limited resources in nursing homes is reported as a challenge [38]. *Legionella* prevention is one of several legal requirements put on nursing homes, which may be part of the explanation as to why compliance is low. Performing and updating the assessments is resource labour intensive and requires specific training, especially in complicated building structures; nevertheless, it is crucial to ensure that *Legionella* prevention has a good effect.

### Risk assessments lead to water management programmes

Our results suggest that the risk assessment is advantageous for further preventive work, possibly due to heightened awareness and/or higher expertise. This is supported by the fact that over 96.5% have introduced a *Legionella* prevention programme following a risk assessment, as opposed to some 59.6% of the nursing homes without risk assessments, thus further underlining the importance of a risk assessment.

A total of 330 nursing homes have introduced a water management programme, meaning that 102 (24,6%) of the participating nursing homes have done so without the introductory risk assessment. While this is positive, as any measure may be better than none, it may also be challenging. Even though water management plans have some general features in common, the outcome of the risk assessment should tailor critical control points to fit each location [39]. Hence, omitting a risk assessment, or indeed neglecting to update it, may lead to insufficient *Legionella* management and even a false sense of security if the water management programme does not perform as expected, since it is not tailored to specific risks.

More than one-third of the nursing homes (35.5%) with risk assessments had installed a unit for continuous biocide treatment compared to those without risk assessments (4.6%). Although our results indicate that nursing homes performing risk assessments mostly applied a chemical treatment, other treatments such as thermal treatment have also shown to be similarly effective [40, 41]. The reason for preferring a specific preventive method was not part of this research and could be explored further. Even though it should ideally be the case, hot water may be unable to reach all areas at a sufficiently high temperature to have preventive effect [42] and bacteria may also be present in cold water pipes [43]. Opting for hot water flushing seems to be regardless of risk assessment status, as 22.9% without a risk assessment have included it as a preventive measure, compared to 25.9% with risk assessments. This also means that 79 out of 109 (72.5%) locations without risk assessments, and indeed 88 out of 228 (61.4%) with risk assessments, seem to altogether lack a disinfection scheme as part of the water management programme.

To prevent *Legionella* proliferation and to ensure that preventive routines in potable water systems have an effect, removal of dead legs to avoid stagnant water is seen as key. This measure was performed more frequently in nursing homes with risk assessments than those without (44.7% vs 16.5%). Our results suggest that risk assessments contribute to discovering and avoiding areas of stagnant water within the water distribution system.

Monitoring *Legionella* levels is not mandatory according to Norwegian legislation, but in Norway it is a highly recommended part of any water management programme in high-risk facilities such as nursing homes. In total, approximately 60% of the nursing homes in this study state that they have a monitoring scheme. Again, the risk assessment seems to be relevant, with over 67% of the facilities with risk assessments doing monitoring, compared to just under 24% of the ones without a risk assessment. This fits with the assumption that risk assessments serve as a good basis for a water management programme.

### Risk assessment control points vary

Norwegian guidelines provide detailed control points to always be included in a comprehensive *Legionella* risk assessment. Key points include temperature monitoring, mapping of building internal water distribution systems, and identifying risk areas for *Legionella* proliferation and spread. Our results suggest that several critical control points have been omitted; e.g., 86% performed temperature control and less than 50% assessed potential aerosol spread. The rationale for omitting risk assessment control points was not part of our study and needs further investigation, but possible explanations are that nursing homes lack the knowledge to perform a comprehensive risk assessment or lack human or financial resources. Reliable answers also depend on the respondent having extensive knowledge of the details of the risk assessment, which may not always have been the case.

### Limitations

A response rate of under half of Norwegian nursing homes makes it uncertain if the results may be extrapolated directly to the remaining facilities. In addition, the lack of knowledge about the difference between nursing homes that responded to the survey and those that did not, do not allow us to assess whether there is a potential information bias. The lack of response may be due to not all emails being forwarded from the municipal administration to the nursing homes. There is also some uncertainty as to whether the survey was answered by the correct person, i.e., personnel involved in *Legionella* prevention at the facility. Furthermore, we did not collect data on how critical control points, such as temperature measurements, was identified in the risk assessment and applied in the water management programme, which did not allow for a deeper investigation.

## Conclusions

This study provides novel insight into adherence to legislative provisions on *Legionella* control in Norwegian nursing homes. Our results suggest that adherence is limited, specifically on performing the mandatory risk assessment. A significant proportion have introduced a *Legionella*-specific water management programme without performing a risk assessment, which may lead to a suboptimal water management programme. Where risk assessments are performed, it seems to benefit the introduction and design of water management programmes, *Legionella* monitoring, removal of dead legs, and preventive biocide treatment.

The lack of adherence raises the need for increased knowledge and awareness about *Legionella* prevention among Norwegian nursing homes. More research is needed to investigate why nursing homes do not perform risk assessments, and to further clarify to what extent the contents of the water management programmes are based on the risk assessment. Further studies could include interviews to gain a more in-depth understanding of the content in risk management plans and the issues faced by nursing homes in assessing and managing this risk.

### Supplementary Information

Supplementary Material 1.

## Data Availability

Collected data and supplementary material may be obtained upon request to the corresponding author. It has not been made available as it is in Norwegian and may need to be translated to non-Norwegians.
