# Supplementary material for: Adherence to Legionella control regulations and guidelines in Norwegian nursing homes: a cross-sectional survey
Source: BMC Public Health. 2024 Jun 4;24:1491. doi: 10.1186/s12889-024-18993-x (PMC11149300; doi:10.1186/s12889-024-18993-x)
Supplement: Supplementary file 1 — Supplementary Material 1. [file 12889_2024_18993_MOESM1_ESM.docx]

**Supplementary file 1**

**Questionnaire in the study “Adherence to Legionella control regulations and guidelines in Norwegian nursing homes: a cross-sectional survey”**

Questions highlighted in bold are included in the data analysis.

1. Institution name.
   1. Free text reply.
2. Institution type.
   1. Nursing home.
   2. Hospital.
3. **County (replies are a list of Norwegian counties as of April 2021).**
   1. Trondelag.
   2. Vestland.
   3. Agder.
   4. Vestfold and Telemark.
   5. Innlandet.
   6. Viken.
   7. Troms of Finnmark.
   8. Oslo.
   9. Nordland.
   10. Møre og Romsdal.
   11. Rogaland.
4. Municipality.
   1. Free text reply.
5. Where have you acquired knowledge about the *Legionella* bacterium? Multiple replies possible.
   1. NIPH webpages.
   2. Legionella Guidance.
   3. Training.
   4. From colleagues.
   5. External company.
   6. Conferences.
   7. Other.
6. Do you know of the Norwegian guiding document on *Legionella* control?
   1. Yes.
   2. No.
7. How comprehensible is the guiding document in terms of risk assessments in health care facilities?
   1. Very comprehensible.
   2. Comprehensible.
   3. Neutral.
   4. Comprehensible to an extent.
   5. Not comprehensible.
8. How understandable is the guiding document in terms of preventive measures against *Legionella*?
   1. Very comprehensible.
   2. Comprehensible.
   3. Neutral.
   4. Comprehensible to an extent.
   5. Not comprehensible.
9. Do you have input to the current guiding document?
   1. Free text reply.
10. How many incidences of legionellosis due to infection from the internal water system in your institution have occurred during the last five years?
    1. 0.
    2. 1.
    3. 2.
    4. 3 or more.
    5. Unknown.
11. **Have you ever performed a risk assessment for *Legionella*?**
    1. Yes.
    2. No.
    3. Unknown.
12. How often is the risk assessment updated?
    1. Annually.
    2. Less than annually.
    3. Performed only once.
    4. Other.
13. **When was the last year you performed a risk assessment?**
    1. 2019.
    2. 2018.
    3. 2017.
    4. 2016.
    5. 2015.
    6. Prior to 2015.
14. **What control points were included in the risk assessment? Multiple replies possible.**
    1. Temperature control (water boilers, pipes and taps).
    2. Legionella monitoring.
    3. Total plate count.
    4. System description and/or drawings.
    5. Identifying areas at risk of *Legionella* proliferation.
    6. Assessing risk of *Legionella* proliferation in technical instalments.
    7. Assessing exposure potential.
    8. Describing previous preventive actions.
    9. Suggested preventive routines.
    10. Other.
15. **Have you established a system for *Legionella* prevention?**
    1. Yes.
    2. No.
16. If no, why not?
    1. *Legionella* is not present in the internal water distribution system. Multiple replies possible.
    2. Lack of resources.
    3. Lack of knowledge.
    4. The guidance document is incomprehensible.
    5. Unknown.
    6. Other.
17. Who is responsible for the *Legionella* prevention?
    1. Building owner.
    2. Technical staff.
    3. Medical staff.
    4. Unknown.
    5. Other.
18. Who performs *Legionella* prevention tasks?
    1. External company.
    2. External company and internal resources in cooperation.
    3. Internal resources.
    4. Unknown.
    5. Other.
19. Do you monitor *Legionella* levels?
    1. Yes.
    2. No.
20. What method do you use for *Legionella* monitoring? Multiple replies possible.
    1. Plating technique.
    2. PCR.
    3. Unknown.
    4. Other.
21. How often do you monitor *Legionella* levels?
    1. Monthly.
    2. Every three months.
    3. Annually.
    4. Other.
22. What percentage of *Legionella* samples taken in 2019 detected *Legionella*?
    1. Not detected.
    2. 1-10%.
    3. 11-30%.
    4. 31-50%.
    5. Over 50%.
    6. Unknown.
23. **What preventive measures are included in your *Legionella* control programme? Multiple replies possible.**
    1. Preventive water treatment, e.g., hot water flushing or continuous biocidal treatment.
    2. Removal of dead-legs.
    3. Refurbishing the internal water distribution system.
    4. Pipe insulation.
    5. Regular temperature control.
    6. General maintenance.
    7. Cleaning and disinfection of shower heads and hoses.
    8. Flushing of rarely used taps.
    9. Staff training.
    10. Other.
24. How often do you clean and disinfect shower equipment?
    1. Monthly.
    2. Bimonthly.
    3. Every three months.
    4. Every six months.
    5. Annually.
    6. Other.
25. Did you ever detect *Legionella* prior to implementing a preventive programme?
    1. Yes.
    2. No.
    3. Unknown.
26. Do you have an internal water system that is no longer in use?
    1. Yes.
    2. No.
    3. Unknown.
27. What year did you implement a preventive programme?
    1. 2020.
    2. 2019.
    3. 2018.
    4. Prior to 2017.
    5. Unknown.
28. Why did you implement a preventive programme? Multiple replies possible.
    1. As a precaution.
    2. Following legionellosis cases among the users.
    3. Following legionellosis cases among the staff.
    4. Approached by commercial companies.
    5. Rumors.
    6. Courses and training.
    7. After reading the *Legionella* guidance.
    8. After acquiring information from other sources.
    9. Economic reasons.
    10. Implemented in a new building.
    11. Other.
29. **What type of disinfection do you use for *Legionella* prevention? Multiple replies possible.**
    1. Periodic thermal treatment.
    2. Continuous biocidal or UV treatment.
    3. Mechanical filtering.
    4. Unknown.
    5. Other.
30. How is hot water flushing performed? Multiple replies possible.
    1. At 70°C or more, 4 times a year or more.
    2. At 70°C or more, 2-3 times a year.
    3. Temperature measurements during flushing.
    4. Minimum of 5 minutes flushing.
    5. Thermal flushing, but less than 70°C.
    6. Other.
31. What type of continuous chemical disinfection method have you installed?
    1. Anodic oxidation.
    2. Ozonation.
    3. Hydrogen peroxide.
    4. Copper and silver ionization.
    5. UV irradiation.
    6. Other.
32. What type of mechanical barriers have you installed?
    1. Water inlet filter.
    2. Shower head filter.
    3. Other.
33. Does hot water flushing have the desired effect?
    1. Very much so (no *Legionella* detected.
    2. Good effect (some *Legionella* detected).
    3. No effect (no detectable reduction of *Legionella* levels).
34. If not, what is the most probable cause?
    1. Free text reply.
35. Does chlorination have the desired effect?
    1. Very much so (no *Legionella* detected.
    2. Good effect (some *Legionella* detected).
    3. No effect (no detectable reduction of *Legionella* levels).
36. If not, what is the most probable cause?
    1. Free text reply.
37. Does chlorine dioxide treatment have the desired effect?
    1. Very much so (no *Legionella* detected.
    2. Good effect (some *Legionella* detected).
    3. No effect (no detectable reduction of *Legionella* levels).
38. If not, what is the most probable cause?
    1. Free text reply.
39. Does monochloramine treatment have the desired effect?
    1. Very much so (no *Legionella* detected.
    2. Good effect (some *Legionella* detected).
    3. No effect (no detectable reduction of *Legionella* levels).
40. If not, what is the most probable cause?
    1. Free text reply.
41. Does anodic oxidation have the desired effect?
    1. Very much so (no *Legionella* detected.
    2. Good effect (some *Legionella* detected).
    3. No effect (no detectable reduction of *Legionella* levels).
42. If not, what is the most probable cause?
    1. Free text reply.
43. Does ozonation have the desired effect?
    1. Very much so (no *Legionella* detected.
    2. Good effect (some *Legionella* detected).
    3. No effect (no detectable reduction of *Legionella* levels).
44. If not, what is the most probable cause?
    1. Free text reply.
45. Does hydrogen peroxide treatment have the desired effect?
    1. Very much so (no *Legionella* detected.
    2. Good effect (some *Legionella* detected).
    3. No effect (no detectable reduction of *Legionella* levels).
46. If not, what is the most probable cause?
47. Does copper and silver ionization treatment have the desired effect?
    1. Very much so (no *Legionella* detected.
    2. Good effect (some *Legionella* detected).
    3. No effect (no detectable reduction of *Legionella* levels).
48. If not, what is the most probable cause?
    1. Free text reply.
49. Does UV radiation have the desired effect?
    1. Very much so (no *Legionella* detected.
    2. Good effect (some *Legionella* detected).
    3. No effect (no detectable reduction of *Legionella* levels).
50. If not, what is the most probable cause?
    1. Free text reply.
51. Does ultra filtration have the desired effect?
    1. Very much so (no *Legionella* detected.
    2. Good effect (some *Legionella* detected).
    3. No effect (no detectable reduction of *Legionella* levels).
52. If not, what is the most probable cause?
    1. Free text reply.
53. Does filter shower heads have the desired effect?
    1. Very much so (no *Legionella* detected.
    2. Good effect (some *Legionella* detected).
    3. No effect (no detectable reduction of *Legionella* levels).
54. If not, what is the most probable cause?
    1. Free text reply.
55. Do you have routines to address discrepancies?
    1. Yes.
    2. No.
    3. Unknown.
56. Is there a need for further information or guidance from the Norwegian Institute for Public Health about *Legionella* control?
    1. Yes.
    2. No.
    3. Unknown.
57. If yes, what type of information or guidance? Multiple replies possible.
    1. Extended guidance aimed at health care facilities.
    2. Check lists designed for health care facilities.
    3. Improved general knowledge on *Legionella*.
    4. Improved knowledge on *Legionella* risk assessment.
    5. Improved knowledge on *Legionella* prevention.
    6. Arenas for knowledge and experience exchange.
    7. Other.
